# Vein watershed analysis locational method versus computed tomography-guided percutaneous localization for detecting non-palpable peripheral pulmonary nodules: a real-world study of non-inferiority

**DOI:** 10.1093/icvts/ivae225

**Published:** 2024-12-31

**Authors:** Chen Huang, Zi-Hao Chen, Li-Shan Peng, Jia-Tao Zhang, Jun-Tao Lin, Sheng Zhang, Jie Yang, Ben-Yuan Jiang, Xue-Ning Yang, Wen-Zhao Zhong, Qiang Nie

**Affiliations:** Guangdong Cardiovascular Institute, Guangdong Provincial People’s Hospital & Guangdong Academy of Medical Sciences, Guangzhou, China; Guangdong Lung Cancer Institute, Guangdong Provincial Key Laboratory of Translational Medicine in Lung Cancer, Guangdong Provincial People’s Hospital (Guangdong Academy of Medical Sciences), Southern Medical University, Guangzhou, China; Guangdong Lung Cancer Institute, Guangdong Provincial Key Laboratory of Translational Medicine in Lung Cancer, Guangdong Provincial People’s Hospital (Guangdong Academy of Medical Sciences), Southern Medical University, Guangzhou, China; Guangdong Lung Cancer Institute, Guangdong Provincial Key Laboratory of Translational Medicine in Lung Cancer, Guangdong Provincial People’s Hospital (Guangdong Academy of Medical Sciences), Southern Medical University, Guangzhou, China; Guangdong Lung Cancer Institute, Guangdong Provincial Key Laboratory of Translational Medicine in Lung Cancer, Guangdong Provincial People’s Hospital (Guangdong Academy of Medical Sciences), Southern Medical University, Guangzhou, China; Guangdong Lung Cancer Institute, Guangdong Provincial Key Laboratory of Translational Medicine in Lung Cancer, Guangdong Provincial People’s Hospital (Guangdong Academy of Medical Sciences), Southern Medical University, Guangzhou, China; Guangdong Lung Cancer Institute, Guangdong Provincial Key Laboratory of Translational Medicine in Lung Cancer, Guangdong Provincial People’s Hospital (Guangdong Academy of Medical Sciences), Southern Medical University, Guangzhou, China; Guangdong Lung Cancer Institute, Guangdong Provincial Key Laboratory of Translational Medicine in Lung Cancer, Guangdong Provincial People’s Hospital (Guangdong Academy of Medical Sciences), Southern Medical University, Guangzhou, China; Guangdong Lung Cancer Institute, Guangdong Provincial Key Laboratory of Translational Medicine in Lung Cancer, Guangdong Provincial People’s Hospital (Guangdong Academy of Medical Sciences), Southern Medical University, Guangzhou, China; Guangdong Lung Cancer Institute, Guangdong Provincial Key Laboratory of Translational Medicine in Lung Cancer, Guangdong Provincial People’s Hospital (Guangdong Academy of Medical Sciences), Southern Medical University, Guangzhou, China; Guangdong Cardiovascular Institute, Guangdong Provincial People’s Hospital & Guangdong Academy of Medical Sciences, Guangzhou, China; Guangdong Lung Cancer Institute, Guangdong Provincial Key Laboratory of Translational Medicine in Lung Cancer, Guangdong Provincial People’s Hospital (Guangdong Academy of Medical Sciences), Southern Medical University, Guangzhou, China; Guangdong Lung Cancer Institute, Guangdong Provincial Key Laboratory of Translational Medicine in Lung Cancer, Guangdong Provincial People’s Hospital (Guangdong Academy of Medical Sciences), Southern Medical University, Guangzhou, China; Department of Thoracic Surgery, Ganzhou Hospital of Guangdong Provincial People’s Hospital, Ganzhou Municipal Hospital, Ganzhou, China

**Keywords:** pulmonary nodule, thoracoscopic wedge resection, three-dimensional reconstruction, locational, watershed analysis

## Abstract

**OBJECTIVES:**

In recent years, with the advancement of sublobar resection, a safe, painless method for locating peripheral pulmonary nodules was needed. Previously, an alternative method of arterial watershed localization was introduced to remedy the shortcomings of preoperative computed tomography (CT)-guided localization or other methods for locating pulmonary nodules, but its technical limitations were discovered during clinical applications. Therefore, we developed a technique to localize non-subpleural nodules using basin analysis of the target vein and validated its feasibility and safety.

**METHODS:**

We performed a retrospective analysis of surgical cases of pulmonary nodules smaller than 2 cm in our centre. The vein watershed locational method (V-WALM) was compared with CT-guided percutaneous puncture localization wedge dissection in terms of success rate, the mean duration of the operation, mean volume of intraoperative bleeding and median postoperative stay, mean postoperative drainage and mean drainage tube indwelling time.

**RESULTS:**

V-WALM and CT-guided localization were used for localized resection of pulmonary nodules in 50 patients. The localization success rates were 94.0% for V-WALM and 90.0% for CT-guided localization, respectively, with no statistical difference noted. In addition, no statistical difference in patient population distribution between the 2 groups was noted. The operating time was 95.5 ± 26.4 min for V-WALM and 94.3 ± 37.5 min for CT-guided localization, with no statistical difference. Neither were there statistical differences in intraoperative bleeding, postoperative drainage and drainage tube indwelling time. The lymph node sampling rate of V-WALM was 48.0%, which was much higher than the 24% noted in the CT-guided localization group.

**CONCLUSIONS:**

The results of this study demonstrate that V-WALM is a safe and feasible intraoperative localization method for peripheral lung nodules. It provides a high-precision, fast and minimally invasive approach to intraoperative localization.

## Graphical Abstract

**Figure:**
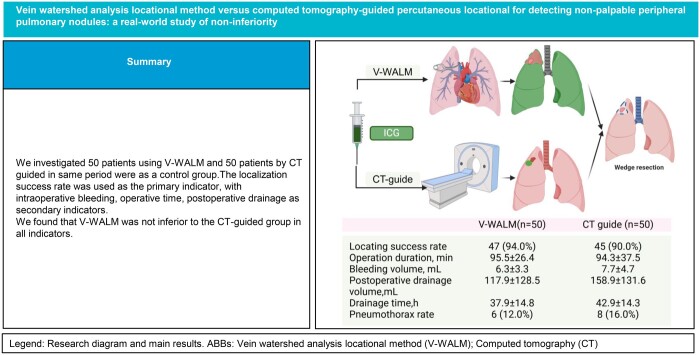

## INTRODUCTION

Statistics show that lung cancer ranks second among all cancers in terms of global incidence and is the leading cause of cancer-related deaths [1]. Recent advancements in medical technology, such as low-dose computed tomography (CT) for early lung cancer diagnosis and screening, have effectively reduced the rate of lung cancer-related deaths [2]. An operation is the primary treatment approach for early-stage pulmonary nodules, and it has been demonstrated to improve patient prognosis [3–5], with previous research particularly focusing on minimizing surgical trauma, expediting patient recovery and ensuring treatment efficacy.

Other researchers have suggested that sublobar resection is more suitable for stage IA non-small cell lung cancer when the tumour diameter is <2 cm. For stage IA1 non-small cell lung cancer, wedge resection is not inferior to segmentectomy in terms of patient prognosis [4, 6–8]. However, for stage IA2 tumours, segmentectomy offers a greater advantage in terms of recurrence-free survival, despite its higher surgical complexity and longer recovery period [9, 10].

Pulmonary nodules are often concealed beneath the pleura; direct intraoperative localization proves challenging, with finger palpation or instrument sliding achieving a mere 30% success rate [11] and intraoperative ultrasound localization yielding limited results [12]. Currently, CT-guided percutaneous placement of markers or injection of biological dyes, though commonly used, carries risks of detachment, displacement, contamination and complications, while also being procedurally complex [13–15]. In addition, near-infrared fluorescence has gradually been used in thoracoscopic surgery [16]. On the other hand, the technical threshold for electromagnetic navigation is high [17–19].

Our team proposed the intraoperative real-time localization method for watershed analysis [20], which was promoted and applied [21]. However, the artery-watershed locational method (A-WALM) is limited by the anatomical complexity of the arterial vasculature in some lung segments and the underdeveloped fissures. Therefore, a simpler intraoperative lung nodule localization method is needed. We innovatively proposed using the more superficial veins as target vessels with 3-dimensional (3D) reconstructed maps to localize pulmonary nodules.

The goal of this study was to propose a more minimally invasive method for V-WALM lung nodule localization. We analysed and summarized the feasibility and safety of this method and compared it with CT-guided percutaneous puncture localization in patients with V-WALM.

## METHODS

### Patients

A total of 50 patients who underwent V-WALM between July 2022 and November 2023 at Guangdong Provincial People’s Hospital in the real world were analysed, and 50 patients who underwent wedge resection after CT-guided percutaneous puncture locating during the same period were included as a control group. Inclusion criteria included size (diameter of lesions between 5 mm and 2 cm); depth (the lesions were located in the peripheral one-third of the lung); and morphology [consolidation tumour ratio (CTR) < 0.5 or considered the nodule to be benign]. The exclusion criteria were (1) chronic obstructive pulmonary disease; (2) iodine allergy; (3) other primary lesions requiring segmentectomy or lobectomy; and (4) having undergone previous ipsilateral lung surgery.

Given the retrospective nature of the study, the requirement for individual informed consent was waived by the ethics review board of our hospital because the attending surgeon explained the surgical plan and risks to the patients before the operation. This study adhered to the principles outlined in the Helsinki Declaration and received approval from the ethics committee of Guangdong Provincial People’s Hospital (No. XJS2023-022-01).

### Preoperative evaluation

**Figure 1:**
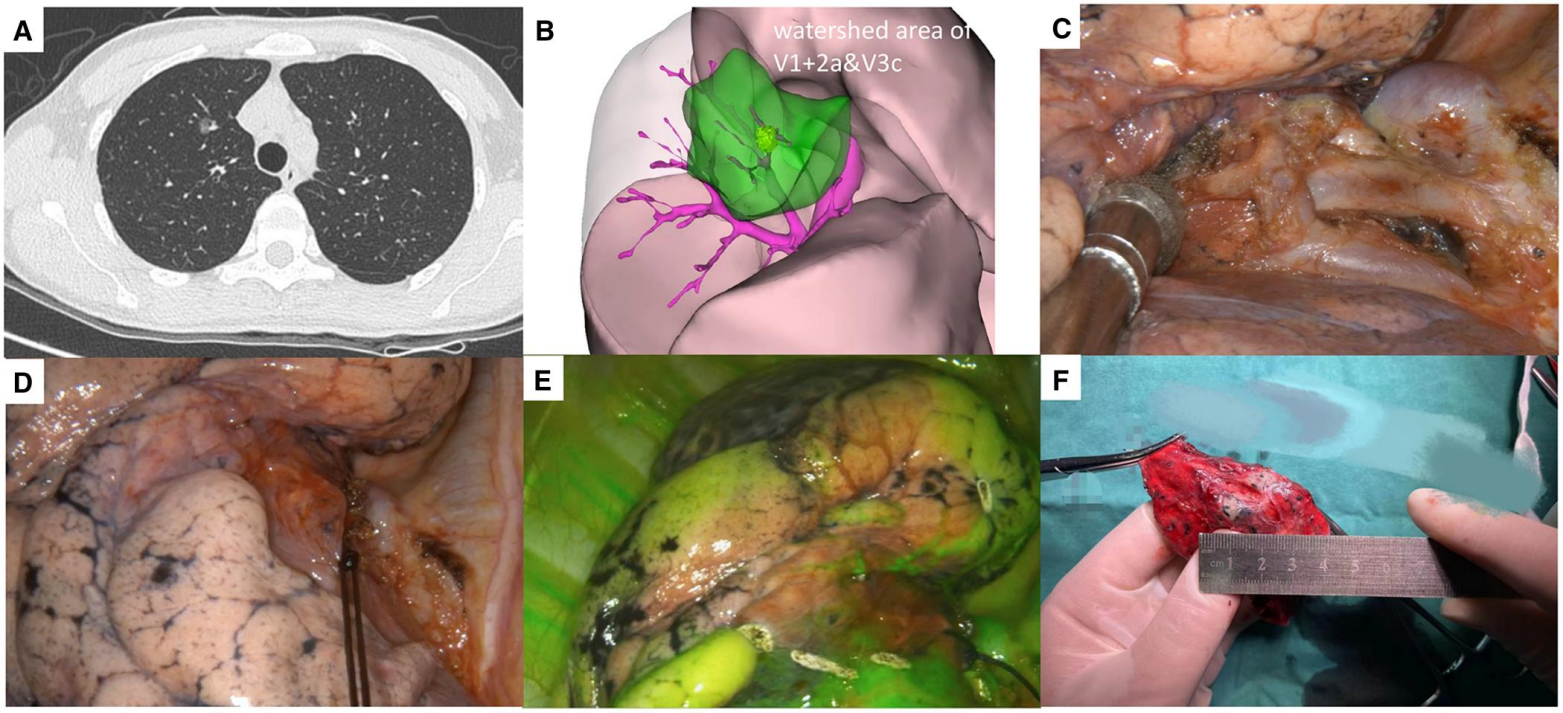
Operative process for the vein watershed locational method. **(A)** Preoperative computed tomography scan to determine the location of the nodule; **(B**) 3-dimensional reconstruction of the left upper pulmonary vein, suggesting that it might be located within the watersheds of V1 + 2a and V3c, and reconstruction of the watershed maps; **(C)** thoracoscopic exploration, dissection and identification of the target vein; (D) blockage of the target vein using a filament thread; **(E)** visualization of the pleura using the fluorescence pattern after injection of indocyanine green into a peripheral vein and marking the watershed boundaries with an electro-hook according to the line of negative stain area; **(F)** resection of the nodule and dissection of the lesion.

Following our centre’s standardized procedure, 64-channel multidetector thin-section chest high-resolution CT examinations (with a slice thickness of 1 mm or 1.25 mm) were conducted (Fig. 1A). Using the CT scan data, we used 3D image analysis software (Materialise mimics 22.0, Materialise NV, Leuven, Belgium) to convert the data into 3D digitally reconstructed, automatically generated 3D reconstructions of pulmonary vessels, bronchial trees and lung parenchyma.

We used the pulmonary segmental vein to locate the nodule as a potential target vein for the nodule. Subsequently, the watershed of the target vein was simulated and analysed by delineating the arteries surrounding the target vein, which was combined with artificial intelligence to simulate the extent of the blood supply of the target vein and extended towards the pleural surface to obtain a projection of the negative-staining area.

Finally, preoperative planning was performed based on the reconstruction results to customize an individualized reconstruction report that included lesion location, pulmonary vein anatomy, identification of target veins that may need to be blocked and the projection area of the target vein watershed (Fig. 1B).

### Computed tomography-guided percutaneous pulmonary puncture localization

After CT scanning, a fine needle was used to puncture the chest percutaneously. The CT scan was repeated to determine the location of the needle adjacent to the pulmonary nodule; then a mixture of indocyanine green (ICG) and melphalan was injected via the puncture needle, and the scan was re-done to determine the location point in spatial relation to the nodule. The patient underwent the operation within 24 h.

### Surgical procedure

After the administration of general anaesthesia, the patient was positioned laterally. The surgeon made a 3- to 4-cm incision between the ribs along the anterior axillary line. Utilizing video-assisted thoracoscopic surgery, the targeted vein was dissected according to a 3D reconstruction map (Fig. 1C). If desired, the watershed lymph nodes can be biopsied incidentally during the dissection of the veins.

Following dissection of the target vein, it was temporarily blocked with a bulldog clamp, a colour clip or a 7-gauge silk slider, depending on the surgeon’s preference. If the nodule is located in the centre of the watershed area, the target vein may be selectively severed (Fig. 1D).

Subsequently, 2 ml of ICG (dissolved in 10 ml of sterile water for injection) was rapidly administered via a peripheral vein. Then, the thoracoscopic camera needs to be switched to the infrared mode. When this is done, the target vein watershed area remains unstained by ICG, whereas the watershed boundaries are visible on the pleural surface (Fig. 1E).

Ultimately, guided by the 3D reconstruction and intraoperative markings, the surgeon performed a wedge resection using staplers, ensuring that the resection margin exceeded the tumour diameter (Fig. 1F). Additionally, each surgical procedure was video-recorded to provide comprehensive quality control documentation.

### Statistics analysis

This study integrated and analysed research variables using descriptive statistics. Specifically, continuous data were presented as means (standard deviations) and medians (interquartile ranges), whereas categorical data were displayed as counts (percentages). Continuous variables were tested using *t*-tests, and categorical variables were analysed using Pearson’s χ^2^ test. In addition, we analysed the applicability of each target vein by descriptive statistics.

The primary study indicator was locating success rate, defined as the availability of a clear intraoperative locating spot and a wedge resection that could be a guided wedge resection by the method only (without the need for additional lung segment resection for remediation). Secondary indicators included operating time, intraoperative bleeding, postoperative drainage, drainage tube indwelling time and lymph node biopsy rate.

All data were analysed using IBM SPSS Statistics version 24 (IBM-SPSS, Armonk, NY, USA).

## RESULTS

From July 2022 to November 2023, a total of 50 patients (14 males and 36 females; median age 54 years; range 29–77 years) were included in the experimental group, and 50 patients (16 males and 34 females; median age 52 years; range 25–78 years) were included in the control group. Patient characteristics and surgical information are summarized in Table 1. There was no statistically significant difference in the population distribution of patients.

**Table 1:** Clinical information about patients undergoing the vein watershed locational method and and those undergoing computed tomography-guided wedge resection

Patient characteristics	Number (%) of patients		*P*
	V-WALM (*n* = 50)	CT-guided wedge resection (*n* = 50)	
Gender			0.391
Male	14 (28.0)	16 (32.0%)	
Female	36 (72.0)	34 (68.0%)	
Median age [range], years	54 [29–77]	52.1 [25–78]	0.411
Mean nodule size, mm	11.1 ± 4.2	9.6 ± 3.5	0.065
Mean depth of nodule from pleura, mm	10.1 ± 6.4	11.7 ± 7.8	0.277
Nodule number in watershed area			
Solitary	49 (98.0)	–	
Multiple	1 (2.0)	–	
Radiological pattern			0.172
Pure ground-glass nodule	15 (30.0)	22 (44.0)	
Mix ground-glass nodule CTR < 0.5	22 (44.0)	14 (28.0)	
Mixed ground-glass nodule CTR > 0.5	9 (18.0)	11 (22.0)	
Solid nodule	6 (12.0)	3 (6.0)	

CT: computed tomography; CTR: consolidation tumour ratio; V-WALM: vein watershed locational method.

Of the 50 patients, 47 successfully underwent intraoperative pulmonary nodule localization using the V-WALM, a locating success rate of 94%. A total of 45 cases were successfully located using CT-guidance, a locating success rate of 90%.

Most patients underwent resection of a single nodule, whereas 4 patients in the test group required simultaneous removal of 2 nodules compared with 3 patients in the control group. One patient had both nodules excised within the same venous watershed area. Nodules were categorized as pure ground-glass nodules (GGNs) in 15 cases (30.0%) in the test group and in 22 cases (44%) in the control group; mixed GGNs with a CTR < 0.5 were identified in 22 (44.0%) of the V-WALM and in 14 (28%) of the CT-guided cases; mixed GGNs with CTR > 0.5 were noted in 9 (18.0%) patients, and solid nodules were noted in 6 (12.0%) of the V-WALM and 11 (22%) and 3 (6%) of the CT-guided procedures. Based on the CT scans, the average diameter of the resected nodules of the V-WALM was 11.1 ± 4.2 mm, with an average depth from the pleura of 10.1 ± 6.4 mm, compared with a 9.6 ± 3.5 mm diameter and 11.7 ± 7.8 mm depth for the CT-guided resected nodules. There was no statistical difference in the distribution of any of the patients’ clinical information.

Regarding surgical outcomes, the mean operative duration for the 47 successful V-WALM cases was 95.5 ± 26.4 min, and the mean intraoperative blood loss was 6.3 ± 3.3 ml. No patients experienced an allergic reaction to iodine during the peripheral intravenous ICG injection. Pneumothorax, defined as >20% lung compression on an X-ray scan, was diagnosed in 6 patients (12.0%) on postoperative day 1. The average chest tube indwelling time was 37.9 ± 14.8 h, with a postoperative drainage volume of 117.9 ± 128.5 ml. The median hospital stay was 3 days (range: 2–5 days) (Table 2).

**Table 2:** Surgical information of patients undergoing the vein watershed locational method and those undergoing CT-guided wedge resection

Patient characteristics	Number (%) of patients		*P*
	V-WALM (*n* = 50)	CT guide wedge (*n* = 50)	
Location success rate	47 (94.0)	45 (90.0)	0.461
More than one wedge to perform	4 (8.0)	3 (6.0)	0.695
Pathological diagnosis			0.132
Adenocarcinoma in situ	6 (12.0)	7 (14.0)	
Minimally invasive adenocarcinoma	19 (38.0)	29 (58.0%)	
Invasive adenocarcinoma	23 (46.0 )	12 (24.0)	
Benign disease	2 (4.0)	2 (4.0)	
Negative margin rate	50 (100)	50 (100)	–
Sampling rate of lymph nodule	24 (45.0)	12 (24.0)	0.012
Mean operation duration, min	95.5 ± 26.4	94.3 ± 37.5	0.850
Mean bleeding volume, mL	6.3 ± 3.3	7.7 ± 4.7	0.238
Median postoperative stay [range], days	3 [2–5]	3 [2–6]	0.753
Mean volume of postoperative drainage, mL	117.9 ± 128.5	158.9 ± 131.6	0.124
Mean drainage tube indwelling time, h	37.9 ± 14.8	42.9 ± 14.3	0.093
Pneumothorax (compress ≥ 20%) (postoperative day 1)	6 (12.0)	8 (16.0)	0.564
Allergy to iodine	0 (0)	–	–

CT: computed tomography; V-WALM: V-WALM: vein watershed locational method.

The mean operating duration in the CT-guided wedge group was 94.3 ± 37.5 min, and the mean intraoperative blood volume was 7.7 ± 4.7 ml. Eight patients (16.0%) were diagnosed with pneumothorax on review of their radiographs on postoperative day 1. The mean drainage tube indwelling time was 42.9 ± 14.3 h, and the volume of the postoperative drainage was 158.9 ± 131.6 ml. The median postoperative stay was 3 (2–6) days. There was no statistical difference in the surgical information between the 2 groups.

It is worth mentioning that the lymph node biopsy rate was significantly higher in 24 V-WALM cases (48%) compared to 12 control cases (24%).

Among the 50 surgical cases analysed, V-WALM shows different distribution patterns across different lung regions. Upper lobes were the most commonly targeted, with 33 cases (66.0%), likely due to their favourable anatomical structure and vascular distribution. Lower lobes were targeted in 15 cases (30.0%), indicating the suitability of V-WALM in this region. Middle lobes were the least utilized, with only 2 cases (4.0%). At the segmental level, the anterior, apical and apicoposterior segments were the most frequently targeted. Vascular distribution and nodule locations in these segments facilitated precise V-WALM localization. Moreover, the inferior lobe’s posterior segment was targeted in 7 cases (14.0%), underscoring V-WALM’s versatility across lung segments. Regarding subsegmental vessels, V1 + 2a and V3b were most commonly used, with 6 (12.0%) and 8 (16.0%) cases, respectively (Table 3).

**Table 3:** The distribution of the vein watershed locational method performed in each lung segment and the distribution of the use of each target vein by the vein watershed locational method

Lobes	Target segment vein	Target subsegment vein	Number of cases (percent)
Upper lobe			33 (66.0)
	V1		7 (14.0)
		V1a	3 (6.0)
		V1b	3 (6.0)
		V1	1 (2.0)
	V2		2 (4.0)
		V2a	1 (2.0)
		V2c	1 (2.0)
	V1 + 2		9 (18.0)
		V1 + 2a	6 (12.9)
		V1 + 2a+b	2 (4.0)
		V1 + 2a & V3c	1 (2.0)
	V3		15 (30.0)
		V3b	8 (16.0)
		V3b+c	1 (2.0)
		V3c	3 (6.0)
		V3	3 (6.0)
Middle lobe or lingular segment			2 (4.0)
	V4		1 (2.0)
	V5		1 (2.0)
Lower lobe			15 (30.0)
	V6		7 (14.0)
		V6a	2 (4.0)
		V6b	5 (10.0)
	V7, V8		2 (4.0)
	V9 + 10		1 (2.0)
	V10		5 (10.0)
		V10a	1 (2.0)
		V10	4 (5.1)
Total			50

## DISCUSSION

A total of 47 V-WALM lobectomies were successfully performed among 50 patients. The results of this study validate V-WALM as a surgical locating modality that is not inferior to CT guidance, uncovering its remarkable advantages in lung anterior segments, apical segments, lingular segments and lower lobes, thereby offering a more precise surgical strategy for clinical practice.

Compared to conventional sublobar resection techniques, a traditional segmentectomy or a combined subsegmentectomy, while effective, is associated with substantial trauma, slow postoperative recovery and elevated risk of complications, thereby increasing the burden on the patients [9, 22, 23]. Direct palpation for nodule localization is limited by nodule location, size and solid components, resulting in low success rates (only 30%) [11]. Intraoperative ultrasound localization is affected by the degree of lung atrophy [12].

CT-guided localization, despite its technological advancement, involves complex procedures, significant radiation exposure, notable pain and positioning accuracy constraints due to factors such as bony structure obstruction and pleural thickening, accompanied by non-negligible risks of complications like pneumothorax, haemothorax and pleural reactions. In the context of strained medical resources, coordinating CT resource utilization poses an additional challenge [13, 14, 16]. As a control group in this study, the 5 cases of CT-guided failures included 2 cases of dye dispersion and 3 cases in whom the accuracy of localization of deeper nodules resulted in wedge resections that failed to find the nodule and required extended segmental resection for remediation. Additional remedies also increased mean operative duration and intraoperative haemorrhage. Moreover, CT guidance requires the borrowing of CT equipment from the radiology department. This process also takes an additional 10–15 min. So it seems that CT positioning is even at a disadvantage in terms of the timing of the full process.

In recent years, the concept of anatomical lobectomy has gained widespread acceptance, particularly the segmentectomy that simplifies bronchial/venous disconnection steps, significantly expediting surgical procedures while adhering to oncologic principles. Otherwise, ICG, a tracer tightly bound to plasma proteins, has been innovatively applied for lymph node visualization [24]. The use of fluorescent endoscopes with ICG has become more common in various surgical procedures [25]. By administering a peripheral ICG injection after pulmonary artery occlusion, a phenomenon of “out-segment staining and in-segment non-staining” (negative stain), which preliminary trials have shown to be comparable to the inflation–deflation method but more rapid [26].

Our team has pioneered the watershed analysis and localization method for precise positioning of pleural-invisible small pulmonary nodules, a technique widely recognized in China [20, 21]. It involves blocking rather than severing arteries, leveraging preoperative 3D reconstruction and the relationship between the target artery’s reverse staining area and nodule location to achieve intraoperative real-time localization, preserving more healthy lung tissue after wedge resection. This method simplifies procedures, enhances accuracy, reduces localization difficulty and avoids discomfort from preoperative CT localization and trauma from segmentectomy.

However, the arterial watershed analysis method faces challenges: The A3 and A10 arteries are located in the deep venous/bronchial surfaces, with long and obstructed pathways, making them difficult to free. Up to 74% of patients with incomplete fissure development require fissure splitting and therefore incur more trauma [27].

**Figure 2:**
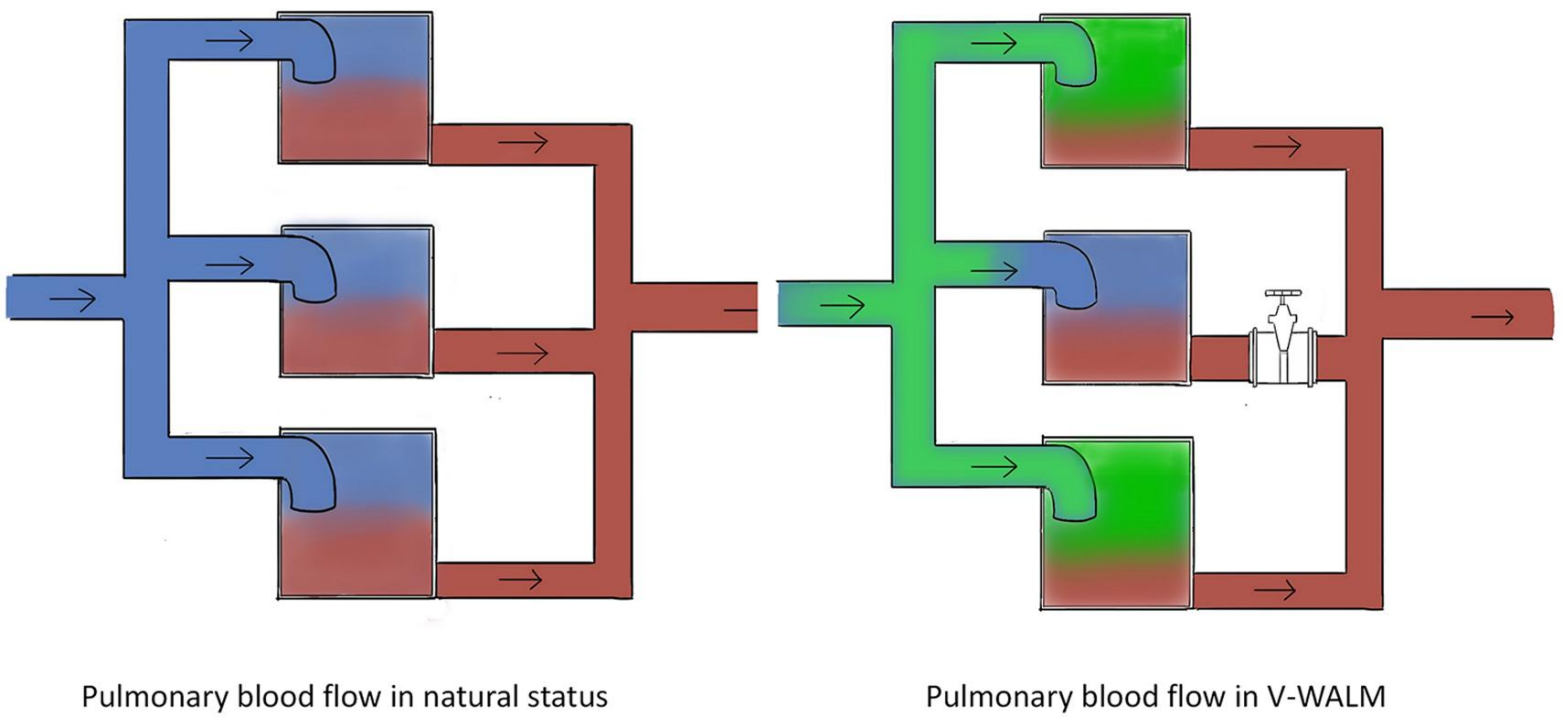
Schematic diagram of the basic principles of the vein watershed locational method. V-WALM: vein watershed locational method.

To compensate for the limitations of arterial watersheds, we attempted a preliminary investigation of the feasibility of using more superficial veins as target vessels. A study by Zhang *et al.* revealed differences in intersegmental planes after venous cutoff, whereas Xu *et al.* [19, 28] suggested that 3D reconstruction of segmental veins could determine these planes, offering new insights into venous watershed feasibility. Contrary to the direct blood flow occlusion in the arterial method, the venous approach produces reverse staining through altered blood flow pressure. Blocked areas experience hindered blood flow reflux, raising capillary pressure above segmental artery pressure, and hindering ICG entry into local pleural surface vessels (Fig. 2). It has been observed that stable boundary formation occurs faster when using the venous watershed method (1–1.5 min) compared to the arterial method (3.5 min) [29]. However, 1–1.5 min is sufficient to complete border labelling.

V-WALM exhibits greater minimally invasive advantages over A-WALM. The anatomical distribution of pulmonary arteries deep within the hilum complicates pure arterial dissection, especially for some segmental arteries, posing higher surgical risks due to increased tissue damage and potential bleeding points. In contrast, most pulmonary veins of the lung segments are relatively superficial except for the central vein, which simplifies the difficulty of target vessel dissection, reduces surgical risk and promotes faster recovery. When compared with data from the A-WALM study previously reported from our centre [20], meaning bleeding volume was reduced from 12.9 ± 9.7 ml in A-WALM to 6.3 ± 3.3 ml in V-WALM, meaning that the operating duration was shortened from 142.6 ± 52.8 min to 95.5 ± 26.4 min in A-WALM, and mean drainage tube indwelling time was not significantly different between the A-WALM 35.6 ± 20.0 h and the V-WALM 37.9 ± 14.8 h, and the lack of postoperative drainage flow in the A-WALM study makes comparison impossible. The V-WALM of 37.9 ± 14.8 h was not significantly different, and the A-WALM study lacked postoperative drainage information; therefore the 2 methods could not be compared. However, the postoperative pneumothorax rate of 26.9% was much higher than the 12% in V-WALM.

The ability of V-WALM to avoid the inherent shortcomings of A-WALM may be due to anatomical differences in the pulmonary arteries/veins: First, manipulation of the oblique fissure can be avoided when freeing the inferior pulmonary vein, which originates independently from the inferior pulmonary hilar. Second, the alternating distribution of V/A-WALM areas provides different pathways for the localization of complex nodules and increases the precision of watershed topography. Segments like the anterior/lingual/basal segments present greater anatomical challenges for arterial dissection, but they have V-WALM advantages for easy venous isolation and higher target vessel utilization. In contrast, the V2/V8 watersheds are more challenging and correspond to more accessible A2.asc or A8.

The choice between arterial and venous localization requires a combination of patient-specific considerations, surgical needs and arterial and venous advantages. For example, in preoperative planning for S1a nodules, the V1a and A1a watersheds are considered, and intraoperatively, easier-to-manipulate vessels are selected.

In summary, suitable target vessels should be tailored according to nodal characteristics and patient conditions, and the flexible use of arteries and veins maximizes surgical benefits and patient recovery.

Although the V-WALM has demonstrated remarkable advantages, we must prudently acknowledge its accompanying limitations and challenges. The surgeon needs to know the distribution of the veins well, especially the inferior pulmonary veins, which can be easily confused. Moreover, the precise execution of V-WALM also depends on high-resolution 3D reconstruction technology. In addition, the shorter time of the V-WALM negative stain, combined with the possibility of mixing in watershed areas (which we hypothesized might be related to bronchial artery perfusion or the degree of carbon deposition on the pleural surface), made the marking of borders less clear in some cases.

In addition to technical shortcomings, even though not observed clinically, we remain alert to potential complications of V-WALM, such as the increased risk of thrombosis of the venous stump after segmental resection.

Both A-WALM and V-WALM have their respective limitations and blind spots. Given the differing scopes of A-WALM and V-WALM, individualized preoperative planning tailored to nodule locations is essential for the precise selection of target arteries or veins. Due to the unique nature of watershed analysis-based localization, it is difficult to directly conduct randomized controlled trials comparing the merits of arterial and venous approaches. Consequently, the conclusions of this study are derived primarily from comparative analyses found in previously published articles.

Furthermore, these conclusions need to be investigated further in prospective studies with larger sample sizes using segmentectomy, A/V-WALM and other localization methods to confirm the safety and utility of V-WALM.

## CONCLUSION

V-WALM provides surgeons with a more convenient and non-inferior method to perform a CT-guided percutaneous lung puncture for the intraoperative real-time localization of lung nodules. Complementing each other with A-WALM in their respective applicable lung segments, WALM becomes a more minimally invasive and non-blind method for localizing lung nodules. This new localization method needs to be further validated through the application of more cases in subsequent clinical practice.

## Data Availability

The data underlying this article cannot be shared publicly because of the need to protect the privacy of individuals who participated in this study. The data will be shared on reasonable request to the corresponding author.

## ABBREVIATIONS

A-WALM: artery watershed locational method
CT: computed tomography
CTR: consolidation tumour ratio
GGN: ground-glass nodule
ICG: indocyanine green
3D: 3-dimensional
V-WALM: vein watershed locational method
